# Involvement of Male Partners in Skilled Birth Care in the North Dayi District, Ghana

**DOI:** 10.1155/2019/2852861

**Published:** 2019-07-01

**Authors:** Farrukh Ishaque Saah, Elvis Enowbeyang Tarkang, Joyce Komesuor, Eric Osei, Evelyn Acquah, Hubert Amu

**Affiliations:** ^1^Department of Epidemiology and Biostatistics, School of Public Health, University of Health and Allied Sciences, Hohoe, Ghana; ^2^Department of Population and Behavioural Sciences, School of Public Health, University of Health and Allied Sciences, Hohoe, Ghana; ^3^Institute of Health Research, University of Health and Allied Sciences, Ho, Ghana

## Abstract

**Background:**

With more than half of the global maternal deaths occurring in sub-Saharan Africa, skilled attendance during childbirth is essential in achieving safer births and lower maternal mortalities. Given that societal ascriptions of gender roles strongly influence the utilisation of skilled care by women, male partner involvement in skilled birth is essential. We explored male partner involvement in skilled birth at the North Dayi District of Ghana.

**Methods:**

This qualitative study interviewed 14 mothers and their male partners, together with two health professionals. The participants were purposively recruited using in-depth interviews. Data collected were analysed manually, but thematically.

**Result:**

Male partners had inadequate knowledge of childbirth and the skilled birth process as well as possible complications arising during delivery. Even though the male partners demonstrated positive perception towards skilled birth and their involvement in the process, their actual involvement in skilled birth care was generally low. Factors which inhibited most of the male partners from getting involved in skilled birth care were health facility nonconduciveness and occupation. However, motivations to do so were marital commitment and sense of responsibility, past experience, nearness to health facility, and safety and survival of partner and baby.

**Conclusion:**

These findings imply that Ghana may not be able to meet the Sustainable Development Goal Three target of reducing its maternal mortality ratio from 216 to below 70 per 100,000 live births by 2030. Stakeholders in Ghana's health industry need to develop male accommodating skilled birth policies and approaches to promote male involvement in skilled birth care.

## 1. Introduction

The health of a woman during pregnancy and childbirth is essential in reducing maternal mortality and achieving the Sustainable Development Goals (SDGs) [1]. Inherent in goal three of the SDGs is a reduction in the global maternal mortality ratio from 216 per 100,000 live births in 2015 to below 70 per 100,000 live births by the year 2030. The goal is expected to be achieved by ensuring universal access to sexual and reproductive healthcare for all, especially women in their reproductive age. An important aspect of sexual and reproductive healthcare which the goal seeks to achieve is skilled birth [1].

Skilled birth is crucial in ensuring that women obtain quality care and deliver in a setting that is equipped for emergencies [2]. It is seen as a key strategy in the reduction of maternal complications and death [3]. During childbirth, the presence of a skilled birth attendant lessens the possibility of stillbirth or death resulting from complications such as sepsis, eclampsia, and obstructed labour by about 20% [3]. Globally, one in every four births (25%) occur without the assistance of a skilled birth attendant. This results in over 40 million unattended births, particularly in developing countries [3].

Societal ascriptions of gender roles for men and women strongly influence access to skilled birth care for pregnant women [4]. The gender division of roles in households usually makes male partners the heads of their households. Being the heads of their households, the male partners usually decide when and what means of accessing healthcare the family including the women should use [5]. Male partner involvement refers to “the various ways in which men relate to reproductive health problems and programmes, reproductive rights, and reproductive behaviour” ([6], p. 1). Male partner involvement in maternal healthcare also refers to the direct assistance provided by men to improve their partners' and children's health through the perinatal, antenatal, labour, and delivery period [7].

Notwithstanding the fact that male partner involvement plays a key role in the health outcomes of women, many societies and cultures treat pregnancy as solely a women's issue and this may have contributed to men not being invited to learn about and engage in matters related to women's and children's health [8]. Universal reproductive health programmes and services in developing countries have also over the years focused on education of women on maternal healthcare such as skilled birth [9]. Yet, in many of these societies, the decision-making authority regarding when and where to seek skilled birth care is vested in the male partner [9, 10]. Men are dominant in the decision-making process in the household [11]. Hence, women are left with little or no say in matters that affect their own lives, including their reproductive health such as delivery care [11].

Similarly, in Ghana, decisions regarding childbirth are the sole vesture of the male partner. Yet, programmes such as sexual and reproductive health education including care and support during pregnancy and childbirth have over the years mainly focused on women [9, 12]. Even efforts of the free-exemption policy for maternal care programme and Safe Motherhood Initiative geared towards involving men in maternal care and skilled birth are yet to be accepted by the populace [13]. This is because there are still deep-seated perceptions regarding male involving in maternal healthcare which posit that male partners have no active role to play in issues concerning delivery care [14].

In this study, the operational definition for the concept of male partner involvement in skilled delivery care was adapted from Kumar [6] and Ampt et al. [7] as responsibility of men with their partners in all aspects of the skilled delivery process and direct assistance provided by men to improve their partners' and children's health through the perinatal, antenatal, labour, and delivery period, respectively. The focus of male partner involvement in skilled delivery was, therefore, based on their readiness to provide emotional, financial, and physical support and accompany their female partners to facility as well as companying them during skilled delivery period.

The role of men in ensuring the safety of their partners during delivery cannot thus be overemphasised. Many studies have indicated positive impacts of male partner involvement in several reproductive health issues [15, 16]. It is important that the relationship between male partner involvement and skilled birth is also investigated to help increase skilled birth utilisation by pregnant women. There is, therefore, a need to ascertain the correlates of male partner involvement in skilled birth care in Ghana, hence this study.

## 2. Conceptual Framework

**Figure 1:**
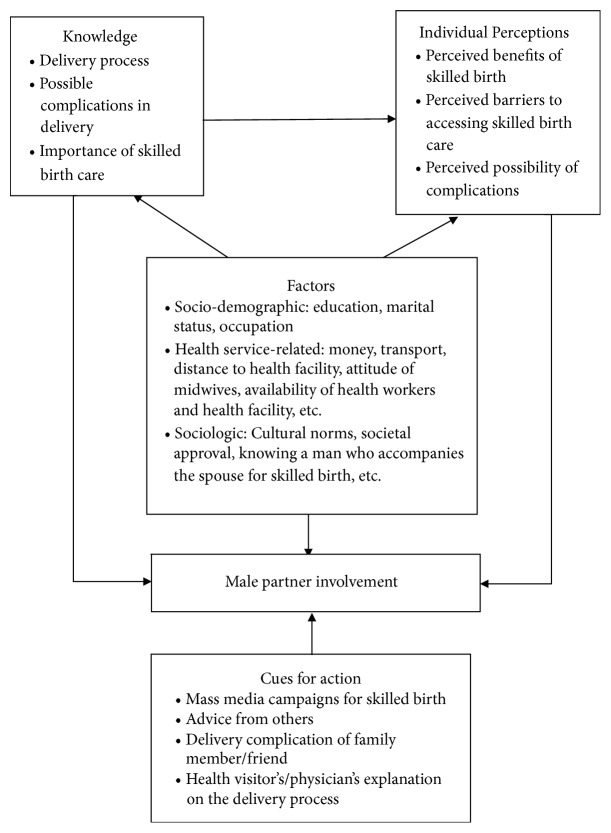
Conceptual framework. Source: adapted from Current Nursing (2012) and Andersen and Newman [18].

The study adapted two theories, Health Belief Model (HBM) and Healthcare Utilisation Model (HUM), as the conceptual framework. This was in recognition of the fact that both models have tenets which are very relevant to the study and compatible with the objectives of the study. Male partners' perceptions about skilled birth and their involvement in skilled birth process are, for instance, posited by the Health Belief Model. The perceived possibility of any complication arising during the delivery process influences the need to seek skilled birth care while comprehending the benefit of delivery by skilled attendant, as argued by Rosenstock [17] in relation to perceived threats (comprising perceived susceptibility and perceived seriousness) of a health condition. A male partner will therefore recognise the need to be involved in skilled birth if he realises that the threat posed by the delivery process to his partner and unborn child's life is severe (Figure 1).

Both the HBM and HUM recognise the role of knowledge in influencing male partner involvement in skilled birth care. Knowledge about the delivery process and skilled birth care as posited by the sociopsychological variables of the HBM, for instance, affects the kind of perceptions a male partner may have about skilled birth and his involvement in skilled birth care. It is also seen by the enabling factors of the HUM as having direct implications for male partners' involvement in the delivery process in that, as they gain knowledge about the process, their appreciation of the need to be involved also increases.

This study also sought to explore the factors influencing male partner involvement in skilled birth care, which the HUM groups into three categories: sociodemographic (predisposing) factors, health service-related (enabling) factors, and sociologic (reinforcing) factors. These factors shape the roles male partners play in the delivery process such as planning for transportation, blood donation if needed, birth preparedness, and supporting the spouse emotionally and psychologically.

The cues to action from the HBM are also relevant to the factors contributing to male involvement in skilled birth care. Mass media campaigns about skilled birth and its importance, advice from other members of the community, and death or some other form of complications from the delivery process of a family member or friend influence male partners' involvement. Also, health visitor's or a physician's explanation of the potential complications associated with delivery process to male partners as well as how skilled attendant at birth reduces chances of complications during delivery invariably affects their involvement in skilled birth.

The framework shows two possible outcomes of male partner involvement in skilled birth. First of all, it indicates that high male partner involvement stemming from knowledge of delivery process results in positive perception towards skilled birth care and involvement manifested through such actions as physical and emotional and financial support. This will lead to good maternal health outcomes while the opposite is true for low male partner involvement resulting from low knowledge, negative perceptions, and persistent factors which act as barriers (Figure 1).

## 3. Materials and Methods

### 3.1. Setting

North Dayi is one of the 25 administrative districts in the Volta Region of Ghana [19]. It is bounded in the north by Kpando Municipality, in the south by South Dayi District, in the east by Afadzato South District, and in the west by the Volta Lake. The 2010 Population and Housing Census of Ghana reports that North Dayi has a population of 39,913, of which females constitute 53.3 % [19]. The Total Fertility Rate for the district is 3.3 births per woman. The General Fertility Rate of the district is 95.0 births per 1,000 women aged 15-49 years. North Dayi has one mission hospital (Anfoega Catholic Hospital), the only top-level facility, and four Community-Based Health Planning and Services (CHPS) zones made up of 23 CHPS compounds. It also has seven (7) health centres with 46 outreach points for rendering Reproductive and Child Health Services [19]. The district has many highlands, creating many hard to reach communities, with very poor road conditions. This makes accessibility to health facilities difficult especially for such communities.

### 3.2. Study Population and Design

This study was qualitative in nature, adopting an explanatory design. This design was adopted in order to identify the extent and nature of cause-and-effect relationship in relation to male involvement in skilled birth care [20]. The target population for this study comprises women attending CWC at Anfoega Catholic Hospital, their male partners, and midwives who assist the women during skilled birth.

### 3.3. Procedures

A purposive sampling procedure was used to identify women attending the Child Welfare Clinic (CWC) at the Anfoega Catholic Hospital. The male partners of the women were then traced to their homes. This was done by selecting the women based on the fact that they had undergone labour and delivery within the past five years. The purpose of using women attending CWC was to ensure that all participants have experienced the central phenomenon of this study, skilled birth. The midwife in charge of the maternity unit of the hospital and another midwife were also purposively included in the study. In all, 14 women attending CWC and their male partners as well as two midwives were included, making a total sample size of 30 participants.

Data for the study were collected by the researcher with the support of two research assistants who were trained for two days to orient them on the purpose of the study and to acquaint them with the research instruments. Women attending Child Welfare Clinic were contacted after their individual sessions and the purpose of the study was explained to them. Those who agreed to participate were included in the study and then led the interviewers to their male partners to also be included. Appointment was booked with the health professional and the interview conducted at her place of convenience, the facility.

In-depth interviews via face-to-face interview procedure were used in collecting data from the participants using three in-depth interview guides. The interview guides were self-developed and used as the instruments for data collection from the three study population groups, women attending CWC, male partners, and the health professionals. Follow-up questions (probes) were asked when necessary in order to obtain detailed insight. The interviews were tape-recorded and notes taken to guarantee accuracy of data that will be collected. Each interview lasted for about 35 minutes.

### 3.4. Data Analysis

Data collected from the participants were analysed using manual thematic analysis. All the recorded interviews, including notes, were transcribed and those not in English were translated and transcribed into English. After this, the transcripts were read and edited by listening to the tapes again and resolving possible omissions and additions in the original transcripts. Codes were combined to form broader themes based on the research objectives as well as themes which emerged from the data itself. Quotes from the participants were used in presenting the data so as to substantiate issues discussed. A frequency table was, however, used to present the sociodemographic characteristics of the participants. Also, level of male partner involvement was determined by comparing roles of male partners played towards and during the skilled delivery period with the operational definition of male partner involvement in skilled delivery care as adopted for this study [6, 7].

### 3.5. Ethical Issues

Ethical approval for the study was granted by the Ghana Health Service Ethical Review Committee (Reference number: GHS-ERC: 63/05/17) before the study was conducted. Permission was taken from the District Health Management Team administration, community leaders (including chiefs and assembly members), and management of the hospital. Written consent was obtained from the participants before including them in the study. This study also ensured the highest level of confidentiality and anonymity in information disclosed to us.

## 4. Results

### 4.1. Sociodemographic Characteristics of Participants

Table 1 presents the sociodemographic characteristics of the 30 participants for this study. Of them, 46.7%, purposefully selected, were mothers, while 6.6% were health professionals and the rest were the male partners. The health professionals were a senior staff midwife and a medical doctor in charge of obstetrics. The participants were aged from 24 and 46 years. A comparative majority (43.3%) were in their late 20s while those who were 40 years and above constituted 6.7%. About 60% were cohabiting and 40% were married at the time of the study. About 73.3% of the participants were Christians and the rest were Muslims. Most of the participants were Ewes (70%) while Guans had the least representation with 10%. Level of education among the participants ranged from JHS to Degree.

### 4.2. Male Partners' Knowledge of Childbirth and the Skilled Birth Process

#### 4.2.1. Knowledge of Childbirth and the Delivery Process

Responses from the female participants and their male partners as well as the health professionals point to the fact that male partners did not have adequate knowledge of childbirth and the delivery process. Concerning male partners not having or demonstrating having knowledge of childbirth and delivery process, a 22-year-old mother said,* “My partner did not do anything to show that he knows something about childbirth and the processes involved in it”.* A male partner, 33 years, also said, “*I don't have much knowledge on it (skilled birth), and I don't need to know because the doctors are there to help us*”.

#### 4.2.2. Knowledge of Complications Occurring during the Delivery Process

Most of the male partners did not have adequate knowledge of the possible complications during delivery. A few of them were, however, aware of vaginal bleeding, prolonged labour, sepsis, swollen hands and feet, placenta retention, eclampsia, breached delivery, stillbirth, and the need for Caesarean section in some instances as some of the complication emanating from childbirth and the delivery process. Regarding male partners not having knowledge of possible complications that can arise during delivery, one of the health professionals, 29 years, for instance said, “*Not all of them are aware of the possible problems. Only a few of them do”*. A 32-year-old male partner also opined,* “No. I don't have any knowledge on this (complications occurring during delivery)”*. A mother, 24-year-old, also noted, “*He was able to identify when I lost appetite for food and got worried, but he doesn't know of any of the possible complications that can occur*”.

### 4.3. Perception of Male Partners Regarding Skilled Birth and Their Involvement in the Delivery Process

#### 4.3.1. Perception of What Constitutes Male Partner Involvement

There were similarities in the way the women and their male partners perceived male partner involvement in skilled birth care. To both groups, it entails the man being present, providing support, and making provisions to ensure safe delivery, as well as making decisions as the head of the household, but in connection with ensuring a safe child birth, paying bills and providing money for feeding during the period. A 27-year-old mother, for instance, said, “*I think he is supposed to be beside me, to feel for me and to tell me ‘sorry dear' in the period of labour pains”*. Among the males, a 37-year-old partner stated,

I understand it (male partner involvement) to mean that the man should be present and take care of the woman during childbirth. It also implies accompanying your wife to the hospital and providing financial support to ensure the baby is born safely. Even making provisions for blood if she needs it during delivery process is my responsibility at that crucial time.

– Male partner, 37 years

The health professionals corroborated the perceptions of both the mothers and the partners regarding what male partner involvement in skilled birth care is. One of them noted,

It (male partner involvement) is about the woman's husband or spouse taking part in the labour and delivery process of their baby by involving himself in all the activities of the delivery process and providing the needed care and support to the woman.

– Health professional, female, 29 years

#### 4.3.2. Perception of Skilled Birth

All the participants acknowledged that the male partners perceive skilled birth care as the best option for delivery. The majority linked their perceptions to the preparedness of the health facilities to attend adequately to complications that may arise during delivery. This view was expressed in the following quote:

Yes! it is good. Sometimes, in the house, they cannot take care of the woman because we, the house people don't know what is going on in the lady. But doctors or nurses at the hospital will know and do something else to make sure the woman and the baby are safe.

– Male partner, 46 years

A 24-year-old mother also said, “*He (her male partner) sees delivery at home as dangerous compared to delivery at the hospital. Home delivery does not have anything to detect complications like baby not turning right*”. Even though the health professionals agreed that male partners perceive skilled birth as a better option compared to home delivery, they also added that some male partners have the view that a person who had previously given birth at home did not have to seek skilled birth care in subsequent deliveries. One of them opined,

They see it (skilled birth) to be the best option as compared to the home delivery especially because of the infections and complications that occur during delivery. For instance, today we had a case of a lady delivering at home before coming with bleeding. Some of them, however, think that when the lady has delivered at home before, it is not necessary for the lady to delivered at the hospital but sometimes it is not the best.

– Health professional, female, 29 years

#### 4.3.3. Perception of Male Partner Involvement in Skilled Birth Process

Participants were asked questions which sought to elicit their perception of male partners' involvement in skilled birth care. To the participants, male partners being involved in the delivery process is a good thing and should be encouraged. One of the health professionals, for instance, had this to say:

I think it is an essential component of the delivery process and I think it really helps in the delivery. Most women who come with their partners you are able to see the difference between those who do not come with their partners. When it comes to finances, especially. When the partners are there, anything you ask they provide for. Otherwise they delay us in calling on the partner to get those items. Even though some of them their partners give them money, it almost always not enough. So, when the partner too come it shows that they are really prepared.

– Health professional, female, 29 years

A mother also said,

I see that his (male partner's) involvement is good and not time wasting as some people would want to make it look like. This is because when there is a problem during the delivery it is all his burden and so, if he is there with me, it makes things easier for me and he is also able to see what is going on.

– Mother, 38 years

A male partner had this to say:

It is a good thing that male partners get involve (in the skilled birth process). Sending someone else to lead her to the hospital or be with her, that person cannot get the needs of my wife that will be requested by the doctors and the nurses done the way I would want and do it. For instance, referrals will require my support to find a car or make the decision and the person may not be able to make that decision for me.

– Male partner, 46 years

### 4.4. Level of Male Partner Involvement in Skilled Birth

Generally, male partner involvement in skilled birth care was low. More than half of the women and their male partners, respectively, as well as the health professionals noted that the male partners were mostly not involved in the delivery process. They either delegated their roles of accompanying and staying with their partners to a female relative or limited those roles to providing financial support and decision-making regarding where the women should give birth. Regarding the fact that male partners were mostly not involved in the skilled birth process, a male partner had this to say:

I did not do much in the delivery process. Besides, the only thing I was supposed to do which I actually did was to give them money for everything needed for the delivery to take place successfully and the hospital bill.

– Male partner, 32 years

A mother also noted,

He was not involved at all. He only gave my sister money and asked her to take me to the hospital. In fact, he left for his work place. I did everything by myself and with my sister's support.

–Mother, 28 years

One of the health professionals remarked,

Their (male partners) involvement in the delivery process is very low in this area. Most of the men do not do anything else apart from providing money for the woman to buy things needed for the delivery process.

– Health professional, female, 29 years

Regarding male partners deciding the choice of place of delivery during the last birth, a 26-year-old mother said, “*It was my husband's decision for me to give birth at the hospital*”. A male partner, 28 years, reported,* “As the man of the house, I was the one who took the decision for her (his female partner) to give birth at the hospital*”. One of the health professionals also noted,

I think it is the husbands/partners who mainly take the decision regarding where the women should give birth when it is time (for the woman to give birth). We, the health professionals, also influence their decision. I think the health professionals help because they listen to us.

–Health professional, female, 29 years

### 4.5. Factors Influencing Male Partner Involvement in Skilled Birth Care

It emerged that factors which influenced male partner involvement in skilled birth care fall into two main categories. While some served as motivators for the involvement of a few of the male partners in the delivery process, others served as barriers inhibiting most of them from getting involved in the process.

#### 4.5.1. Motivations for Male Partner Involvement in Skilled Birth Care

The major motivational factors for male partner involvement identified were marital or relationship commitment and male partner's sense of responsibility, past experience, partner's education and occupation, safety and survival of partner and baby, and nearness to health facility.

Concerning show of affection and relationship commitment, the male partners stated that it was because of the commitment they have towards their partners that they maintained the level of involvement during their partner's last delivery while the mothers interpreted that to mean show of love. A male partner noted,

I believe that once you're married, there are some things that you need to do to show affection towards your partner. If you love your wife, you have to do these things for her. From my elders, it is something a man should do and that is why I supported her.

– Male partner, 46 years

Regarding male partner's responsibility, the mothers whose male partners supported them said their partners considered it their responsibility to be involved. A 24-year-old mother, for instance, said, “*He knew it was his responsibility that was why. He knew that I was not working so he was supposed to take care of everything*”.

With regard to the safety of the mother and baby, both mothers and their male partners indicated that male partners were motivated to be involved because they wanted to ensure the safety and survival of the pregnant woman and baby. A 28-year-old mother, for instance, summarised her view by saying, “*He did not want any problems to befall us (pointing to herself and her child) for him to bear the cost”*. A male partner also indicated,

I saw it as she is someone's daughter so I had to make sure nothing happens to her to be held responsible. Because I know something can go wrong during the delivery. I just needed to make sure that nothing bad happens to them.

– Male partner, 46 years

Regarding nearness to a facility, a participant explained that his involvement was motivated by the fact that the hospital is close to their residence and made it easier for him to be present. The 28-year-old male partner said, “*We are close to the hospital. As you can see, our house is just opposite the hospital and that made it easy for me to monitor everything that was going on throughout the delivery”.*

#### 4.5.2. Barriers to Male Partner Involvement in Skilled Birth Care

The major factors which were proffered as barriers hindering male partner involvement in skilled birth care were health facility nonconduciveness, cultural norm for females to handle childbirth issues, and male partner's occupation. Concerning the nonconduciveness of health facilities, some of the male partners argued that a major reason why they were unable to be with their spouses was the fact that they were not allowed by health providers to be present during delivery. The health facilities were also cited as not being conducive for men and hence their unwillingness to involve themselves. A mother, 28 years old, for instance, said, “*At times, the men are not allowed by the health staff to witness the process even though they want to be part”.* A 32-year-old male partner also retorted, “*We get ignored when we come with our partners to deliver. The midwives will not allow any man to come near the delivery process. They ask us to stay away”*. The health professionals corroborated these views with one of them saying,

The delivery room is not suitable for male partners to be present with us. Generally, per our delivery rules, they don't accept men to be in the labour room since other women may be giving birth at the same time and we can't allow the men to be looking at other people's wives. Some of the men know so they would not want to come at all. Our labour room does not allow them to be in. Even the female relatives, in only serious situations such as the woman being “stubborn” that we allow the relatives to come and talk to her.

– Health professional, female, 29 years

Concerning cultural norm of female relatives handling childbirth issues, participants explained that their involvement in the skilled delivery process was perceived negatively due to the cultural norm that issues surrounding delivery and childbirth are the domain of females. Also, only female relatives are expected to be present and playing direct roles such as assisting the partner in labour. The following quotes present their views:

We grew up to understand that during labour, men are not to be present. Women are supposed to be the only people observing and assisting the woman in labour. So, it is the mother or mother in-laws, aunties and sisters who help the woman. Ours (men) is to just be patient and be ready to provide any ‘manly help' such as carrying her when there is no transport, if the need arises.

– Male partner, 36 years

Traditionally, men are not supposed to be physically present as that is seen as the role of older women in the family such as the mother and mother in-law and sometimes the pregnant woman's sister. The childbirth issues are seen as ours and the only expectations of men is to give us (the women) money for birth items and payment at the facility.

– Mother, 34 years

A male partner, 36 years, also explained that in some instances, due to this cultural norm, men receive undesirable reactions from their mates and relatives which prevented them from involving themselves even if they want to. He said,

When you want to even do something like pack her bag with the items (birth preparedness items) and carry it along while accompanying them, they say a lot of discouraging statements like ‘why do you want to follow? Are you a woman or the midwife?' So, I just let them go”.

Regarding male partner's occupation, most of the participants indicated that the stressful nature of the jobs and the long hours needed by the male partners to put into such works inhibited their ability to get involved in the delivery process. A 28-year-old mother, for instance, said, “*My partner's work could not give him the chance to be with me as I wanted”*. A male partner also argued,

It is because of my work; being a welder, I couldn't absent myself from work to attend to my wife even though I knew her expected delivery is that week. If it was not my work, I think I would have done more.

– Male partner, 32 years

### 4.6. Improving the Level of Male Partner Involvement in Skilled Birth Care

The study also asked the participants to proffer suggestions to improve the level of male partner involvement in skilled birth care. Suggestions given included improved partner communication about childbirth and the delivery process, education and counselling by health workers, and institutional change.

The male partners and the mothers generally suggested that education and counselling by health workers and other opinion leaders can improve male partner involvement in skilled birth care. For instance, a 46-year-old male partner said,

“The health workers at the health facilities pregnant women attend ANC (antenatal clinic) have to be educating the males. There is the need for education so that men can acknowledge what the partner goes through so that they can support”.

– Male partner, 46 years

A mother also added her voice saying,

Men need to be educated and counselled for them to know the importance of they getting involved and the fact that we (the women and the unborn child) need them to survive. Most of them don't know why they should be involved more than they are doing. As a matter of fact, they feel like they are doing everything even just provision of money. The health workers can do that for us.

– Mother, 28 years

Some of the mothers suggested that improved communication between couples and respect for male partners by the women will improve male partner involvement in skilled birth care. In arguing for the need to improve couple communication, a mother, 27 years, noted, “*I should be able to tell my husband what I am going through and what is being told at the antenatal so that he can be more participative”*.

A 22-year-old mother however, suggesting respect for male partners to improve their involvement, said, “*Women should respect their husbands so that they can support them during delivery”*.

Though the health professionals also agreed to the need for education and counselling, they further suggested that health facilities have to change their delivery policies to be more accommodating and friendlier to male partners. One of them noted,

Education. I think the health professionals should educate the men especially in the communities. You won't get them anywhere except the communities. Any man who comes with his wife to ANC for instance, that person is seen before the other people. This encourages them so that they don't spend so much time at the facility. It should be re-enforced to encourage them to be involved throughout the pregnancy and delivery process.

– Health professional, female, 29 years

A male partner also said,

I would have loved to be with my wife in the room while our baby was coming out so that I can encourage my wife to push. That would have possibly prevented the need for operation (Caesarean section) in the last delivery. But the nurses (midwives) said the facility does not allow men to go into the delivery room. I think the nurses should allow us to if we want.

– Male partner, 28 years

## 5. Discussion

Male partners in our study did not have adequate knowledge of childbirth and the skilled birth process. This finding is consistent with previous studies by Nanjala and Wamalwa [21], Salam et al. [22], and Tweheyo et al. [16] who found that male partners have limited knowledge of safe motherhood including skilled birth and the delivery process. The finding of this study, however, contradicts those of Katiso and Adinew [23] and Nasreen et al. [24] who argued that male partners are knowledgeable about childbirth and the delivery process. The fact that male partners were not knowledgeable about childbirth and the delivery process in the present study only reinforces the general assumption in the Ghanaian context that males have nothing to concern themselves with when it comes to issues of maternal and child health. Such issues are considered the sole preserve of women and, as such, men have no need to worry themselves about them. A male who involves himself in the delivery process is typically referred to as a weak man as argued by Abass, Sakoalia, and Mensah [25] and Davis et al. [8]. In relation to the conceptual framework, Andersen [26] argued that knowledge is a factor in adopting a health behaviour or utilising a healthcare service. Hence, the lack of adequate knowledge of childbirth and the delivery process observed in this study influences the involvement level of the male partners.

The finding that most of the male partners lacked appropriate knowledge of the possible complications during childbirth and the delivery process confirms the outcome of a previous study conducted by Nanjala and Wamalwa [21] in Western Kenya. Nanjala and Wamalwa in their study argued that male partners had low level of knowledge concerning delivery-related complications. In the present study, however, male partners were not aware of umbilical cord prolapse and amniotic fluid embolism as complications.

Male partners' lack of knowledge about the possible complications arising during childbirth is argued by Rosenstock [17] in the conceptual framework of the study to imply a lack of perceived threat whose existence would have influenced male partners to be involved in skilled birth if they realise that the threat posed by the delivery process to their partners and unborn children was severe. The fact that male partners had low knowledge about possible problems during childbirth can also be attributed to the fact that maternal issues have been considered to be women's domain. Hence, most men do not see the need to seek the prerequisite knowledge as they do not see its usefulness since they have left these matters to the women, as indicated earlier [27, 28].

The meanings of male partner involvement expressed by the participants agree with those found by Olugbenga-Bello et al. [29]. Olugbenga-Bello et al. argued that the men they surveyed perceived involvement in childbirth and skilled birth process as taking care of their spouses and the unborn/newborn child and providing emotional and moral support as well as providing financial support. The views of participants in the present study also conform to the definition that male partner involvement in skilled birth care comprises combined responsibility of male partners through increased awareness and support for their partner's maternal health needs (Waltson, 2005; [6, 7]). These findings are relevant to the propositions by Andersen and Newman [18] and Rosenstock [17] in the conceptual framework that what is considered male involvement is derived from the knowledge and beliefs the participants upheld. Hence, they defined male partner involvement in delivery care in terms of how much they understood childbirth and perceptions about their involvement.

The finding of positive perception among male partners towards skilled birth in the present study is in line with postulations of Mangeni et al. [30] and Yargawa and Leonard-Bee [31] in which the authors realised that their male partner participants generally had positive perception. The findings are, however, in disagreement with that of Wai et al. [32] which stated that male partners do not perceive skilled birth care positively.

Regarding male partners exhibiting positive perceptions towards skilled birth, this may be attributed to the perceived benefits of seeking skilled birth as against home delivery. In the framework underpinning this study, Rosenstock [17] posits that male partners will assume or favour a particular health behaviour, that is, skilled birth, if they perceive some form of benefit or protection from adopting it. And so, it can be adduced that the perceived benefit from skilled birth is the cause of the positive perception male partners have about it.

Concerning positive perception of male partners about their involvement in skilled birth found, this study is consistent with those of Tweheyo et al. [16] and Olugbenga-Bello et al. [29] which observed that male partners have favourable perceptions towards their involvement in skilled birth. The findings support the conceptual framework's perceived susceptibility of partner to delivery complications which may make the male partner perceive supporting her as important. Perceived benefits of male partner involvement and use of skilled birth care are influential in the level of involvement of male partners in the skilled birth process as argued by Rosenstock et al. [33] and this probably informed the perceptions of the male partners towards their involvement in the process.

The level of male partner involvement realised in this study can be linked to their perception and level of knowledge of the process. From the conceptual framework, Andersen [26] argued that the level of male partner involvement is a result of male partners' knowledge about the skilled birth process. Thus, as observed in this study, almost all male partners lacked adequate knowledge of childbirth and the delivery process and this may have accounted for their low involvement as they do not know what is required of them in the process and what they can do to support their partners during the process. Furthermore, Rosenstock [17] argued that male partners' perceptions are influenced by their knowledge of childbirth and the delivery process and possible complications. This is, however, not the case in this study as most male partners had positive perception about skilled birth and their involvement even though they lacked adequate knowledge of childbirth and the delivery process. Low level of male partner involvement observed in this study is consistent with the lack of knowledge of childbirth and the delivery process though not consistent with the positive perception male partners have of skilled birth and their involvement.

The fact that most male partners included in the present study took part in the decision-making process concerning the choice of place of delivery even though they did not participate in other areas which needed their support (such as accompanying the women to the facility for delivery) could be attributed to the traditional role of men, which is to wield the decision-making power of the household as heads [9, 10]. The findings of the present study, where male partners were the ones who mainly took the decision regarding choice of place of delivery by women, are in line with previous findings by Wai et al. [32] and Dickson et al. [5]. The authors in these studies realised that most men were involved in the decision-making regarding the choice of delivery, supporting the argument that men control decision-making in the home including that of the partner's health. Contrary to this finding, however, was a study by Kwambai et al. [34] which argued that men seldom contribute to the decision regarding maternal care including choice of place of delivery.

Our finding regarding some male partners seeing their involvement as a responsibility leading to their involvement is consistent with the study of Vermeulen et al. [35] which proffered that male partner's perceived role is a factor behind their involvement in skilled birth care. Also, Rosenstock [17] in the conceptual framework argued the fact that male partners' role is a direct function of their perception about the delivery process and what they are to do as men and, for that matter, heads of the family.

Concerning distance to health facility being a factor in male partners' involvement, the participants' responses are congruent with the study by Ditekemena et al. [36] which stated that the closer male partners to the delivery facility, the more the willingness to be involved. Also, Andersen and Newman [18] posited that some health-related factors such as availability of space, health provider attitude, distance to facility, and quality of care are influential in male partners' level of involvement as shown in the framework.

Also, cultural norm of childbirth being the domain of women as an inhibiting male partner involvement in skilled birth care supports the findings of Nanjala and Wamalwa [21] and Abass et al. [25]. In their studies, it was shown that existing cultural norms result in undesirable reactions from family and peers of male partners, which precludes them from participating in maternal processes including skilled birth care. This is further explained by the conceptual framework that cultural norms shape the perception of individuals and subsequently engaging in positive health behaviour like male involvement in skilled birth care.

The finding that delivery facilities were not accommodative for male partners as well as them being not allowed to be present with the partner during delivery is consistent with the studies of Ditekemena et al. [36] and Vermeulen et al. [35]. In their studies, it was found that lack of space to accommodate male partners and the feeling of being ignored by health providers when they accompany their partners for delivery contribute to males' perception that they are not welcomed, thus affecting their involvement and reducing their roles to only finance. Also, this finding is consistent with the study of Kululanga et al. [37] which found that the lack of privacy in the labour room stalled birthing women to have a partner or family member present in the labour ward for emotional support.

The findings point to the important roles of partner's education and occupation in influencing their involvement in issues relating to maternal and child health as realised in previous studies [5, 21]. Regarding employment, Nanjala and Wamalwa note that women with male partners who are formally employed are more likely to seek skilled birth than those with partners who are not employed in the formal sector. In relation to the conceptual framework, Andersen and Newman [18] noted that a person's education and occupation make him or her more willing to assume a particular health behaviour. As such, the involvement of the male partner is a correlate to his available time from work, educational level, and even, to some extent, study area.

Regarding the cues to improve male partner involvement in skilled birth, Rosenstock [17] in the conceptual framework argued that the perceived threat of a health condition can be influenced by some activities to improve the knowledge level of the individual. The participants' suggestion that male partners need to be educated on childbirth and delivery complications as well as the roles they need to play is consistent with the argument that knowledge of the risk of negative maternal and child health outcome and the seriousness of these complications if male partners do not get involved [33] is important.

We relied on verbal reports of behaviour in relation to male involvement in skilled birth. This has the tendency for the interviews to overreport good behaviour as participants may want to provide socially desirable responses. Also, recall bias could have been present as pregnancy may not represent as much significance to the male partner as it does to the female. Nevertheless, the study used both partners and, hence, was able to overcome this by cross-validation of the responses provided in both interviews.

## 6. Conclusion

Male partners' level of involvement resulted more from their sense of responsibility than a general understanding and acceptance of the idea of assisting their partners' maternal health needs. Also, male partners' lack of adequate knowledge of childbirth, the delivery process, and related complications implies that men cannot recognise delivery-related complications and plan together with the partner to seek early medical attention to avert the problem. This will affect the struggle of the country to attain the SDG goal three of reducing maternal and infant mortalities. Stakeholders need to develop a skilled birth policy that encourages male partner presence during labour and delivery as a way of integrating them into the process.

## Figures and Tables

**Table 1:** Sociodemographic characteristics of participants.

Sociodemographic variable	Frequency	Percentage (%)
*Participant Type*		
Mother	14	46.7
Male Partner	14	46.7
Health Professional	2	6.6
*Age*		
20-24	3	10.0
25-29	13	43.3
30-34	7	23.3
35-39	5	16.7
40+	2	6.7
*Sex*		
Male	15	50.0
Female	15	50.0
*Marital status*		
Co-habiting	18	60.0
Married	12	40.0
*Religion*		
Christian	22	73.3
Muslim	8	26.7
*Ethnicity*		
Ewe	21	70.0
Akan	6	20.0
Guan	3	10.0
*Level of education*		
JHS/Middle school	9	30.0
SHS/O' Level/A' Level	17	56.7
Tertiary	4	13.3

## Data Availability

The data used to support the findings of this study are available from the corresponding author upon request.
